# The positive association between number of children and obesity in Iranian women and men: Results from the National Health Survey

**DOI:** 10.1186/1471-2458-8-213

**Published:** 2008-06-15

**Authors:** Enayatollah Bakhshi, Mohammad Reza Eshraghian, Kazem Mohammad, Abbas Rahimi Foroushani, Hojat Zeraati, Akbar Fotouhi, Fraidon Siassi, Behjat Seifi

**Affiliations:** 1 Department of Biostatistics, School of Public Health and Institute of Public Health Research, Tehran University/Medical Sciences, Iran; 2 Department of Nutrition, School of Public Health and Institute of Public Health Research, Tehran University/Medical Sciences, Iran; 3 Department of Physiology, Medicine School, Tehran University/Medical Sciences, Iran

## Abstract

**Background:**

To date, few studies have assessed the association between the number of children and obesity in couples. We aimed to investigate this association in men and women aged 20–75 years.

**Methods:**

Data from the National Health Survey were considered in this investigation. It included 2728 women and men (1364 couples) aged 20–75 years. Height and weight were actually measured rather than self-reported. A generalized estimating equation model was used to estimate the odds of obesity (body mass index (BMI ≥ 30)) as a function of the number of children adjusted for age, sex, education, economic index, workforce, smoking and place of residence.

**Results:**

We infer that each additional child has at least 5% and at most 34% increase in the odds of obesity in men and at least 4% and at most 29% increase in the odds of obesity in women. Our test of interaction by sex showed that the association between the number of children and obesity was not different among men and women. Among women, factors that increased obesity included age, low education, having more children, being inactive workforce and being nonsmoker. Among men, these factors included high economic index, low education, having more children, and being nonsmoker.

**Conclusion:**

Our results show an association between the number of children and obesity among men. We would recommend interventions to reduce the number of children to prevent obesity in men.

## Background

Obesity is considered as the disease of the twenty-first century [1]. In the United States, obesity was not considered as an issue of interest in the mid-1980_s_, but since then, it has become more common: and in 2003–2004, approximately 32.2 percent of the US adult population were obese [2]. Almost one third of adult Canadians are at increased risk of disability, disease, and premature death due to obesity [3]. Obesity is relatively common in Europe, especially in southern and eastern countries, and studies from repeated surveys suggest that the prevalence of obesity has been increasing recent years [1,4]. Excess body weight has been reported to be a risk factor for cardiovascular diseases, diabetes, some cancers and other diseases [5-12].

BMI (Body Mass Index) increases with the increase in the number of children among women [13-15]. This contribution seems to be, on average, less than 2 kg per pregnancy [14,16]. Pregnancy-related weight gain is a significant contribution to weight gain for some women (approximately 15%–20%) [17].

Most studies have identified several factors that could be involved in the weight gained after pregnancy [18-20]. The strongest factor for retaining weight after pregnancy seems to be the weight gain during pregnancy [20,21].

Smoking cessation, which fortunately often occurs once women learn of their pregnancy, will also make women retain more weight [21]. More sedentary lifestyles after pregnancy also appear to be a risk factor [22-24] and socio-economic factors are also associated with higher weight retention after pregnancy [24-26].

In addition, physiological changes can explain the relationship between the number of children and obesity among women, such as hormonal alterations secondary to fewer ovulatory cycles [14], insulin resistance associated with pregnancy [27,28], and increased glucocorticoid activity [29,30] during pregnancy.

Some studies have addressed the association between the number of children and obesity among men. A study on 1039 men aged 50–89 years showed that men with five or more biological children were more obese than men without biological children [31]. In the Rancho Bernardo cohort, a positive relationship between the number of children and obesity among men and women has been observed [32]. In this study, participants were men and women 55 to 84 years old. To date, few studies have assessed this association in couples. Weng HH et al. [33] found a positive relationship between the number of children and obesity among couples. This association was higher in women than in men but not significant. In this study, respondents were couples aged 40–70 years.

We aimed to investigate the association between the number of children and obesity in couples by using cross-sectional data from the National Health Survey in Iran.

## Methods

### Data set examined

The National Health Survey in Iran is a survey designed to gain comprehensive knowledge and information about health care problems and difficulties throughout the country, 1999–2000. Sampling was conducted on the basis of the cluster method, each cluster comprising of 8 households. The choice of 8 households for the cluster size was based on one-day performance capacity of the data collection group: Four persons (2 physicians, 1 interviewer and 1 lab technician). The statistical framework was based on the household lists available with every Health Department in the provinces, usually updated annually. Where household lists were available, selecting the cluster was made systematically. Data from the National Health Survey were considered in this investigation. These data were collected by the National Research Center of Medical Sciences and are presented partially at the Department of Biostatistics and Epidemiology/Tehran University of Medical Sciences for research. We focused our study on the respondents who lived in Tehran Province only, and the analyzed data included 2728 women and men (1364 couples) aged 20–75 years. We excluded pregnant women from the analyses. This study is approved by the Ethic Committee of the Tehran University of Medical Sciences.

### Measurments

BMI (Body Mass Index), our dependent variable, was calculated as weight in kilograms divided by the square of the height in meters (kg/m^2^), and subjects were classified into obese (BMI ≥ 30 kg/m^2^) and nonobese (BMI < 30 kg/m^2^).

Education was defined as the total number of years of education. The respondents were categorized into three groups: those with low (0–8 years), moderate (9–12 years) or high (more than 12 years) education. The three categories used two dummy variables.

The respondents were grouped according to their place of residence as living in cities (urban) or villages (rural). Information about the respondent's age was based on their self-reported birth year.

We had no information on physical activity and alcohol consumption (the consumption of alcohol is prohibited in Iran).

There wasn't any information on household income, but economic index is a surrogate for household income.

Workforce status: The original questionnaire item included twelve response categories. For this analysis, housewife, student, retired and unemployed were grouped into one category named "nonactive workforce" vs. "active workforce" (others).

Economic index was defined as the area of living place (in square meter) divided by number of people in household.

Smoking status dichotomized into smokers (those who smoke every day and have smoked at least 100 cigarettes in their lives) vs. nonsmokers (others).

Information about the number of children was based on self-reports.

### Statistical analysis

Since couples' responses are correlated, the Generalized Estimating Equations (GEE) model, which takes into account the correlated nature of responses, with a logit link and exchangeable correlation structure was used to estimate the odds of a obesity as a function of the number of children adjusted for age, sex, education, economic index, workforce, smoking and place of residence. Economic index, the number of children and age were examined as continuous variables. The other five variables were (0,1) variables. We looked specifically for interactions between sex and all covariates (sex × education, sex × smoking and sex × place of residence were not significant and we excluded these nonsignificant terms). The interaction between sex and number of children was not significant but it is sensible to include a variable that is central to the purpose of the study and report its estimated effect even if it is not statistically significant. A model containing interaction terms implies that both the estimated odds ratios and their corresponding confidence intervals vary as the values of the effect modifiers vary. The results are presented as OR_s _and their 95 percent CI_s_. All analyses were carried out by using SAS software Package, version 9.1.

## Results

Our data included 2728 women and men (1364 couples) aged 20–75 years with a mean age of 36.9 year. The mean BMI of study members was 26.1 kg/m^2^(95% CI: 25.9–26.3); 17.5 percent were obese (BMI > 30 kg/m^2^). The mean number of children and economic index were 2.8 and 21.2 m^2^, respectively. Altogether, 11.6 percent were active workforce. The mean education was 8 years. Of the respondents, 19.5% were smokers, and 93.2% were urban. Table 1 shows the frequency distributions of all variables for men and women by obesity Category.

**Table 1:** Characteristics of the analytical sample by sex and obesity category

Variables	Women			Men		
		
	Obese^a ^(n = 327)	Nonobese (n = 1027)	P-value	Obese^a ^(n = 140)	Nonobese (n = 1224)	P-value
Age, years(mean, sd)	36.1 (7.5)	33.3(7.6)	<0.001	40.4(8.1)	39.8(0.4)	0.45
Number of children(mean, sd)	3.3(1.9)	2.6(1.6)	<0.001	3.1(1.7)	2.7(1.7)	0.04
Economic index(mean, sd)	20.4(11.3)	21.5(12.4)	0.14	22.8(13.2)	21.1(12.0)	0.09
Resident in city (%)	91.7	93.7	0.21	90.0	93.5	0.12
Active workforce (%)	1.8	8.7	<0.001	13.6	16.5	0.37
High education (%)	3.9	8.9	<0.003	10.0	14.4	0.16
Moderate education (%)	28.2	39.6	<0.001	34.3	38.2	0.36
Smoking (%)	1.8	2.4	0.48	30.0	37.6	0.08

^a^Body mass index 30(kg/m^2^) or greater.

We started by fitting a preliminary GEE model including only sex and number of children to observe the influence of the potential confounders on obesity. This model shows that the odds of obesity increased with increasing number of children: unadjusted obesity odds ratios were 1.29(95% CI: 1.21–1.39) and 1.10(95% CI: 1.001–1.20) for women and men, respectively. The unadjusted OR of 1.29 for women was significantly different from the unadjusted OR of 1.10 for men because the interaction between the number of children and sex was statistically significant (p = 0.006).

In GEE model controlling for age, economic index, workforce status, education level, place of residence and smoking status, the odds of obesity increased with increasing number of children: obesity odds ratios were 1.18(95% CI: 1.05–1.34) and 1.16(95% CI: 1.04–1.29) for men and women, respectively. For each additional child, the odds of obesity increased by 18% among men and 16% among women. Our test of interaction by sex showed that the association between the number of children and obesity was not different among men and women (P = 0.79). Comparing two models, we found that the obesity odds ratio in women decreased after adjustment for confounding variables, whereas that for men increased slightly.

Moreover, Table 2 shows that age was directly associated with obesity only among women. Obesity odds ratios were 1.02 (95 percent CI: 1.001–1.05), and 0.98 (95 percent CI: 0.96–1.01) for women and men, respectively. We infer that a 1-year increase in age has 2% increase among women and 2% decrease among men in the odds of obesity.

**Table 2:** Obesity^a ^odds ratios, among 1364 Iranian couples in the GEE analysis, 1999–2000

Independent variables	Odds ratio	95% confidence interval
Number of children (female)^b^	1.16	1.04–1.29
Number of children (male)^b^	1.18	1.05–1.34
Age (female)	1.02	1.001–1.05
Age (male)	0.98	0.96–1.01
Economic index (female)	1.002	0.99–1.01
Economic index (male)	1.02	1.01–1.04
Active workforce (female)	0.25	0.10–0.60
Active workforce (male)	0.76	0.46–1.27
High education	0.60	0.37–0.97
Moderate education	0.76	0.59–0.96
Smoking	0.68	0.48–0.98
Resident in city	0.70	0.47–1.04
Male vs. female: Active workforce^c^	1.21^d^	0.32–4.56
Male vs. female: Nonactive workforce^c^	0.40^e^	0.16–1.00

^a ^Body mass index 30 (kg/m^2^) or greater

^b^The interaction terms were included as cross-products between sex and the predictors of interest(number of children, age, economic index and workforce level) therefore, different OR for male and female were obtained.

^c^Number of children, age and economic index were examined as continuous variables. Male vs. female OR evaluated at the overall mean number of children, age, and economic index.

^d^Among subjects with active level, the odds of obesity for men was1.21 times that for women.

^e^Among subjects with nonactive level, the odds of obesity for men was 0.40 times that for women.

We found a statistically significant association between economic index level and obesity only among men. Obesity odds ratios were 1.002 (95 percent CI: 0.99–1.01), 1.02 (95 percent CI: 1.01–1.04) for women and men, respectively. A 1-m^2 ^increase in economic index has 0.2% in women and 2% increase in men in the odds of obesity.

An inverse association was observed between workforce level and obesity among women. Obesity odds ratios were 0.25 (95 percent CI: 0.10–0.60) and 0.98 (95 percent CI: 0.46–1.27) for active women and men, respectively.

Overall, subjects with lower education were more obese. Using low education as the reference group, obesity odds ratios were 0.76 (95 percent CI: 0.59–0.69) and 0.60 (95 percent CI: 0.37–0.97) for the moderate and high groups, respectively

An association was observed between place of residence and obesity (but nonsignificant). Obesity odds ratio was 0.70 (95 percent CI: 0.47–1.04) for urban couples.

An inverse association was observed between smoking status and obesity. Obesity odds ratio was 0.68 (95 percent CI: 0.48–.98) for smokers compared with nonsmokers.

Among subjects with active workforce level, the odds of obesity for men was1.21 times that for women. In contrast, among subjects with nonactive workforce level, the odds of obesity for women was 2.44 times that for men.

## Discussion

In this cross-sectional study, we assessed associations between the number of children and obesity in men and women. In the first model (without confounders), unadjusted obesity odds ratios were 1.29(95% CI: 1.21–1.39) and 1.10(95% CI: 1.001–1.20) for women and men, respectively. Unadjusted OR of 1.29 for women was significantly different from the unadjusted OR of 1.10 for men. Furthermore, we were able to adjust for covariates that might be important confounders in these associations. After adjustment for confounding variables, number of children is positively associated with obesity in both sexes. We infer that each additional child has at least 5% and at most 34% increase in the odds of obesity in men. For women, the figures are 4% and 29%, respectively. Although this association was stronger in men than in women, the difference was not statistically significant. Our results are basically in line with the observation by Weng HH et al [33].

Comparing two models, the influence of the potential confounders included in analysis can be observed. Unadjusted OR_S _of 1.29 and 1.10 are changed to OR_S _of 1.16 and 1.18 for women and men, respectively. After controlling for potential confounders, the similar associations were seen in both sexes suggest a lifestyle, rather than a biologic, impact of having children in women.

Our findings were consistent with the conclusions of Lawlor et al. [35] and Hardy et al. [36]. Lawlor et. al [35] concluded that "Lifestyle risk factors associated with child-rearing lead to obesity and result in increased CHD in both sexes; biological responses of pregnancy may have additional adverse effects in women". Hardy et al. [36] showed that "Any association between number of children and CHD risk factors is a result of lifestyle and behaviors associated with family life rather than being as result of the biological impact of pregnancy in women".

Our finding may point toward a better understanding of the social and cultural mechanisms of obesity in couples. Most couples seem to be spending more free time with their children. With increasing number of children, couples may have little time to spend on health behaviors. For example, they don't go to the gyms and exercise centers. Children love fast food, and parents have to follow them. Fast food doesn't contain large amounts of fiber, vitamins, minerals, and the like-elements necessary for good nutrition and health. In contrast, these substances can cause or increase obesity. An increased consumption of snacks [37], caloric beverages [38,39] and fast foods [40] by children and young adults has been shown repeatedly to be associated with obesity and excess weight gain.

There are some limitations of our study. This study is a cross-sectional study, which means that we cannot draw definitive conclusions concerning the direction of causality. However, this should be confirmed by further longitudinal studies. Measures of "the age of children" and "the length of time the children lived in the household" are useful in this regard but they were not available for study members.

Our study had several strengths. It was performed in a nationally representative sample of the Iranian couples. Few studies have assessed the association between the number of children and obesity in couples. To our knowledge, ours is the first study in Iran. Height and weight were actually measured rather than self-reported. It is well known that self-reports underestimate the prevalence of obesity [41,42]. This study included couples aged 20–75 years who lived in Tehran province but our findings may be generalized to other provinces as well.

## Conclusion

Number of children is positively associated with obesity in both women and men. For each additional child, the odds of obesity increased by 18% among men and 16% among women. Since obesity is positively associated with cardiovascular disease, cancer, diabetes, and other important causes of morbidity and mortality, we would recommend interventions to decrease the number of children to prevent obesity in men, too.

## Abbreviations

BMI: body mass index; OR: odds ratio; GEE: generalized estimating equation; CHD: coronary heart disease.

## Competing interests

The authors declare that they have no competing of interests.

## Authors' contributions

EB, MRE and KM originated the idea for this study, did the research proposal, data analysis and prepared the manuscript. ARF and HZ co-ordinated the research project. AF helped and edited the final version as the epidemiology consultant. FS helped and edited the final version as the nutrition consultant. BS helped and edited the final as the physiology consultant. All authors read and approved the final manuscript.

## Pre-publication history

The pre-publication history for this paper can be accessed here:


